# Simulation-based education to promote confidence in managing clinical aggression at a paediatric hospital

**DOI:** 10.1186/s41077-020-00139-9

**Published:** 2020-08-12

**Authors:** Marijke Mitchell, Fiona Newall, Jennifer Sokol, Melissa Heywood, Katrina Williams

**Affiliations:** 1 Neurodevelopment & Disability, Royal Children’s Hospital, 50 Flemington Road, Parkville, Victoria, 3052 Australia; 2 Department of Paediatrics, The University of Melbourne, 50 Flemington Road, Parkville, Victoria, 3052 Australia; 3 Murdoch Children’s Research Institute, 50 Flemington Road, Parkville, Victoria, 3052 Australia; 4 Department of Nursing, The University of Melbourne, 50 Flemington Road, Parkville, Victoria 3052 Australia; 5 Nursing Research, Nursing Education, Royal Children’s Hospital, 50 Flemington Road, Parkville, Victoria 3052 Australia; 6 The RCH Simulation Program, Royal Children’s Hospital, 50 Flemington Road, Parkville, Victoria 3052 Australia; 7 Department of Paediatrics, Education and Research, Monash Children’s Hospital, Monash University, 246 Clayton Road, Clayton, Victoria, 3168 Australia

**Keywords:** Simulation training, Child, Patient harm, Aggression, Patient safety, Health personnel

## Abstract

**Background:**

An increasing number of incidents involving aggressive behaviour in acute care hospitals are being witnessed worldwide. Acute care hospital staff are often not trained or confident in managing aggression. Competent management of clinical aggression is important to maintain staff and patient safety. Training programmes for acute care staff are infrequently described in the literature and rarely reported for paediatric staff. Simulation training allows practice of skills without patient risk and may be more effective than traditional teaching formats for aggression management.

**Aim and design:**

The aim of this proof of concept study was to develop a simulation-based education session on aggression management for acute care paediatric staff based on best practice principles, to evaluate the acceptability of this training programme and to gain an understanding of the impact of the training on participants’ perceived confidence in managing clinical aggression. Two separate simulation exercises were delivered as a 2-h component of a hospital management of clinical aggression (MOCA) training day. Participants completed a written survey immediately prior to, at completion of the simulation-based group training, and at 3–6 months following the simulation training.

**Findings:**

Nine training days were conducted in 2017 for nursing, medical, allied health, education and security staff with a total of 146 participants (83% were acute care nurses). Two thirds (68%) of participants had experienced clinical aggression as part of their routine work, with 51% overall reporting a lack of confidence managing these patients. Immediately following the simulation training, 80% of all participants reported feeling more confident in managing clinical aggression, 47% reported a 1-point increase in confidence, whilst 33% of participants reported a 2- or 3-point increase. At 3–6 months post-training, 66% of respondents (*N* = 44) reported continued confidence in managing aggression with 100% of participants stating they would recommend simulation training to colleagues.

**Conclusions:**

Simulation training is an acceptable method of training and shows promise to improve staff-perceived confidence for managing behavioural emergencies in acute paediatric health care settings. In addition, there were potential enduring positive impacts at 3 months after the study. Whilst resource and time intensive, further research assessing the benefits of utilising simulation training in this setting is warranted in order to minimise staff burn-out and improve outcomes for these very vulnerable patients.

## Background

Aggression demonstrated by patients in the acute paediatric hospital setting can result in serious self-injury and threaten the safety of their family, staff and other patients [1]. Clinical aggression episodes in child health facilities warranting a coordinated, systematic response are increasing [2–6]. Paediatric hospital staff, whilst required to regularly demonstrate competence in resuscitation and management of the deteriorating child, are often not trained nor tested in the management of clinical aggression [7–13]. Acute hospital staff report a lack of confidence in managing clinical aggression [7, 8, 10, 14, 15] which, coupled with regular exposure, can lead to high levels of stress and burnout [9, 10, 16]. A lack of confidence in managing clinical aggression can also result in more frequent activations of hospital emergency response systems leading to increased and potentially unnecessary use of chemical and physical restraint and isolation of patients. It is important that staff training methods are effective in increasing staff confidence and skill levels and post-training discussion identifies how to potentially mitigate preventable errors. Paediatric acute health care is a complex specialty encompassing patients with a wide range of developmental levels that operate within an array of unique family centred care systems. Staff need a large set of skills and strategies that can be adapted to effectively interact with the spectrum of ages, developmental stages, neurodiversity and parental/carer involvement experienced in the acute paediatric setting.

Aggression management training programmes for staff working in acute care hospitals are scant [7, 8, 13–21] and rarely described for staff working in the paediatric setting [22, 23]. Programmes vary in format, content and duration hindering direct comparison. Outcome measures are variable in that most increase self-confidence in managing aggression [7, 8, 13, 14] and understanding of how to manage aggression; however, maintenance of skill acquisition is not routinely assessed. Programmes have not been shown to consistently reduce incidents of aggression or restraint [13, 16]. Formats which include practice of skills combined with reflection and reinforcement, such as with simulation-based education, may be more efficacious [18, 24–26].

In order to improve staff skill levels in managing aggression, it would be ideal for staff to practise de-escalation and restraint skills in an environment which closely resembles the environment in which they work [18, 26]. Simulation training allows participants to develop or enhance their knowledge and skills, and analyse and respond to realistic situations in a simulated clinical setting without patient risk [27]. Simulation addresses the growing ethical issues around ‘practice of skills’ on human patients and provides a learning environment where staff can make mistakes, correct errors and rehearse the management of complex and crisis situations without compromising patient safety [25]. Another powerful effect of simulation training may be that it minimises participants from making mistakes in the future by providing them with opportunity to ‘try out’ strategies in a risk-free environment, improve their situation awareness and communication and leadership skills [25]. Simulation training to teach and practice resuscitation and other technical skills is well described in the paediatric acute care literature [28–30]. Similarly, the practice of non-technical skills such as communication techniques is also described in the simulation literature [31–33]. Simulation-based education may be an effective format to practice de-escalation skills for aggressive behaviour [34–38]. However, the use of simulation for teaching management of clinical aggression demonstrated by children and young people in the paediatric acute care setting has not been reported.

## Design and implementation

This is a proof of concept study. The purpose was to design, implement and evaluate a simulation-based group training programme for health professionals which utilises real time training in an environment that closely replicates a clinical ward, with simulated adolescent patients who exhibit aggression whilst accessing hospital services.

The aims of this study were as follows:

- Evaluate the acceptability of this type of training for aggression management for staff for situations that involve typically developing children and young people
- Gain an understanding of the impact of the training on participants’ perceived confidence in managing clinical aggression immediately, post-training and at 3–6 months follow-up.

### Evaluation framework

The Kirkpatrick framework of training evaluation was utilised as it is the most widely used model to assess training [39–41]. It has been used in a number of studies to evaluate the simulation training [42–47] and training focussing on managing challenging behaviour [48, 49]. As this was a proof of concept study to test the acceptability of the training format, the evaluation outcomes were primarily designed to address Kirkpatrick levels 1 and 2 only (reaction and learning, respectively).

### Training design

Clinical aggression is managed in the study hospital via a Code Grey response system. Code Grey is one of a series of emergency response codes used in hospitals in some states of Australia and is activated when aggressive or violent behaviour is identified [50]. This system involves a hospital emergency response to deal with people who are perceived to be or who are actively being aggressive towards another person. At the study hospital, an Australian tertiary paediatric hospital, a response team is activated to attend the incident which is led by a nurse with extensive clinical aggression management experience (Code Grey Coordinator), nurses from three wards and security staff who are all trained in aggression management. The response team works with the local staff to defuse the aggressive incident through verbal de-escalation or restraint.

A 2-h simulation training programme was developed and embedded into the management of clinical aggression (MOCA) training programme at the study hospital from February 2017. This full day MOCA training [17, 51] is conducted by the Code Grey Coordinator and repeated monthly to build the capacity of the hospital-wide response team. Training is available for nursing, medical, allied health, education and security staff who may be involved in the Code Grey response. The course includes lectures on predictors and early warning signs of aggression, crisis assault cycle, communication strategies for managing aggression, legislation and local policies and physical intervention techniques. Annual attendance at MOCA training is mandatory for all staff working in the Mental Health Unit, for those enrolled in the Graduate Nurse Program, and newly employed security staff.

The RCH Simulation Program curriculum and simulation sessions are designed according to the concepts described by Dieckmann et al. [52], utilising the debriefing framework by Rudolph et al. [53]. This simulation-based education session included two separate simulations. The scenarios focused on managing aggression exhibited by a young person in the inpatient setting and were developed by the study investigators in conjunction with the simulation faculty. The simulation scenarios were reviewed, trialled and validated by members of the simulation faculty, with modifications made prior to conducting the sessions to improve the learning experience. Two simulation exercises were developed involving the same patient, a 15-year-old female named ‘Danni’, played by a professional actor (Table 1). Each simulation training session included a 10-min simulation followed by a 30–40 min facilitated debrief. The second scenario utilised the same scenario synopsis and task; however, the level of difficulty was escalated with the simulated patient instructed to react to staff interventions in a more aggressive manner.

**Table 1:** Simulation scenario synopsis

**Synopsis:** Danni is a 15-year-old female with a history of mental health difficulties who was admitted to the Adolescent Unit for investigation of abdominal pain and management. Danni was sitting outside the entrance to the ward with another patient, Ned, playing games on their phones. Both patients were asked to return to the ward by a nurse who went looking for them. Ned returned immediately to the ward whilst Danni became distressed at being told what to do and stormed back to her bedroom. Danni locked herself in the bathroom after slamming the door. **Task:** Convince Danni to open the bathroom door and return to her bed. Use empathy and appropriate communication strategies to calm her and prevent further escalation in her behaviour. **Simulation learning objectives:** By the end of this simulation participants should be able to: • Identify and demonstrate the key communication skills and language required when interacting with a distressed young person. • Maintain safety of the patient. • Demonstrate empathy and insight when communicating with the distressed young person. • Recognise early warning signs of aggression and prevent behavioural escalations • Recognise when it is appropriate to activate a Code Grey response.	

Scenarios were designed to enable participants to primarily focus on applying supportive communication techniques and de-escalation skills to an aggressive situation. To ensure safety of the actor, the simulation was ceased by the simulation faculty if the participants decided the patient required 5-point prone restraint or chemical restraint despite de-escalation measures. Physical and chemical restraint, whilst discussed in the MOCA training day, are not deemed to be first line strategies in managing agitation and increasing aggression for young people in the acute hospital setting and therefore were not incorporated into this simulation training session.

The facilitated debrief explored participants’ frames in order to analyse and reflect on their actions, discussed the communication framework for managing clinical aggression and enabled closure of any gaps in their knowledge.

### Evaluation method

The study design involved delivery of a 2-h simulation-based group training session on the management of clinical aggression to hospital staff with participants asked to complete three surveys: a pre-training survey, post-training survey, and a follow-up survey. The purpose of the pre-training survey was to assess self-perceived levels of confidence and competence in managing clinical aggression. The purpose of the post-training survey was to assess if simulation training had an impact on participants’ self-perception of confidence and competence in managing aggression. The purpose of the follow-up survey was to assess if the simulation training had a continued impact on participants’ self-confidence and competence.

The principal investigator explained the study design to the participants prior to commencement of the simulation pre-brief. The simulation faculty member facilitating the simulation session then briefed participants on the objectives of the two simulation exercises. The roles and expectations of both the participants and the instructors were discussed. The structure of the session and the purpose of the post-simulation debrief was explained. The participants were informed that the same professional actor would play the role of the patient in each of the simulation exercises. The second simulation, whilst utilising the same scenario, was designed to be more complex than the first, providing participants with opportunity to extend their skills and build on knowledge learnt from the first simulation. Participants were asked to volunteer for roles in each simulation scenario. Eight volunteers were required for each scenario: two nurses to be the bedside nurses providing direct care to the patient, two additional nurses working in the ward and three nurses and one security staff member to join the Code Grey Coordinator as the Code Grey response team, if required. Allied health, medical and education staff could also volunteer to be part of the Code Grey team. Staff members were not asked to perform roles outside their scope of practice. After this pre-brief and establishment of participant roles, the simulation technologist orientated participants to the simulation centre and simulation equipment prior to commencement of the first simulation scenario. Participants, who did not have active roles in the first scenario, watched the simulation via live video stream in an adjacent room. Participants changed roles for the second scenario with observers offered the opportunity to be active participants. As group sizes varied from 10–25, not all participants were able to have an active role in one of the simulations; however, all had the opportunity to participate fully in the post-simulation debrief session. The skills required to de-escalate aggression in the simulation scenarios were discussed in the content of the MOCA training day with the simulation training providing an opportunity for participants to apply their knowledge and practice these de-escalation skills.

A short, written survey was administered to the participants’ pre- (survey 1) and post-simulation training (survey 2) to assess acceptability of the training and self-perceived levels of confidence and competence immediately prior to and at completion of training.

A follow-up questionnaire (survey 3) was sent to participants as an electronic survey 3–6 months post-simulation training to assess their continued perceptions of the training and their exposure to aggression in the workplace. The surveys were completed anonymously with no identifying information. Results of the surveys were analysed for each training session and at completion of the study data was combined and analysed as a larger data set. Unique participant identifiers were not used; therefore, survey 1 and 2 results were not able to be linked to the results of survey 3.

### Data security and handling

De-identified hard copies and electronic files will be stored for 5 years in keeping with ethics requirements.

### Study setting and population

The study setting is a tertiary paediatric hospital, The Royal Children’s Hospital, (RCH) Melbourne, Australia.

The study was approved by The Royal Children’s Hospital Melbourne Human Research Ethics Committee (HREC 37142). Health professionals including nursing, medical, allied health and education and security staff enrolled in the hospital MOCA training programme from February–December 2017 were invited to participate in the study. The participants were provided with study information in the form of a verbal script. Written consent to participate in the simulation-based training was gained and participants chose to participate in the study by completing the surveys. It was explained that participants could choose not to complete the surveys without explanation or consequence. In total, 146 staff completed the training in 2017, and it was anticipated that 90% would complete the immediate pre-/post-simulation survey.

Participants who completed the simulation training were invited via email to complete an electronic survey 3–6 months following completion of the simulation training. The survey link was emailed to participants. Two additional reminder emails were sent 2 weeks apart following the initial email. Participants could choose to participate in this part of the study by completing the survey. Participants could choose not to complete the survey without explanation or consequence. It was anticipated that 20% of participants would complete the survey.

All participants who attended the MOCA training were eligible to participate in the study. There were no exclusion criteria as all participants who work at the study hospital speak English and were able to complete the written survey.

The simulation-based education was conducted in the simulation centre using an environmental set-up which was a close replica of a hospital inpatient room, involved hospital staff performing roles similar to their current roles and utilised a trained actor as a simulated patient. As a result of this experience, it was recognised that participants could potentially become distressed during the simulation scenario training session. Supportive processes for debriefing and support were put into place in the event a participant became distressed during the training session.

### Statistical methods

This study employed descriptive statistics, assessment of Likert scale questions and qualitative allocation to themes of written comments for the survey data. Quantitative data used a 5-point Likert scale to assess pre-training self-reported confidence and competence in managing clinical aggression as well as immediately following and 3–6 months following the training. The investigators used proportions of ratings, means, standard deviations and confidence intervals to describe the results. Means were used to analyse Likert data to provide more powerful understanding of the data [54–56]. Changes in self-perceived confidence were compared prior to and following the training using paired *t* tests and reported at a group and individual level. We defined success as 80% of participants reporting an increase in confidence. The post- and follow-up groups were unsuitable for paired *t* test comparisons of mean scores as the two samples were not independent and unable to be linked due to anonymity. The differences in mean confidence scores between the post- and follow-up group were presented in a descriptive manner.

## Evaluation findings

### Participants

Nine Management of Clinical Aggression (MOCA) training courses were conducted in 2017 for 146 participants. Ninety-five percent of participants completed the pre- and post-simulation questionnaires. Close to 40% of participants were graduate nurses who had recently commenced their Graduate Nurse Program, 44% were ward nurses with the remainder of participants a mix of security staff, medical and allied health staff and nurses working in the mental health unit (Table 2).

**Table 2:** Participant demographics

	Pre-training ***n*** (%) ***N*** = 140	Follow-up ***n*** (%) ***N*** = 44
Graduate nurse (GNP)	55 (39)	16 (36)
RN	51 (36)	12 (27)
Clinical nurse specialist (CNS); clinical nurse educator (CNE); associate nurse unit manager (ANUM)	11 (8)	5 (11)
Security staff	9 (6)	1 (2)
Mental health RN	7 (5)	6 (14)
Other	4 (3)	2 (5)
Allied health professional	2 (1)	2 (5)
Medical staff—trainee	1 (1)	0 (0)
Staff <1 year experience	69 (49)	16 (36)
Staff >6 years’ experience	34 (24)	11 (25)
Work in a mental health setting	32 (23)	9 (20)
Have been the recipient of clinical physical or verbal aggression	95 (68)	24 (55)

*CNS* clinical nurse specialist, *CNE* clinical nurse educator, *ANUM* associate nurse unit manager, *RN* registered nurse

Two thirds of participants (64%) had minimal prior exposure to clinical aggression, with 44 (31%) never witnessing a Code Grey, 26 (19%) witnessing one, and 20 (14%) witnessing two Code Greys. One group was more experienced in the management of clinical aggression with 29 (21%) having participated in 10 or more Code Grey activations prior to the study.

### Acceptability

At the completion of the simulation training, participants were asked to indicate how relevant they thought this training was to their work. Most of the participants, 123 (88%), chose a rating of 4 or 5 out of 5 to indicate the level of relevance. All participants stated that they would recommend attendance at a simulated MOCA training session.

### Perceived confidence immediately after training

Participants rated their confidence in managing clinical aggression (1 = not at all confident; 5 = extremely confident) prior to the simulation training. Prior to undertaking training, 76% (*n* = 106) rated their confidence as a 2 or 3 out of 5 with 1 indicating that they are not confident at all and 5 indicating they felt extremely confident. Immediately following completion of the training, 62% (*n* = 87) rated their confidence as a 4 or 5 out of 5.

Immediately after completing the simulation training, 81% (*n* = 113) reported feeling more confident in managing clinical aggression. A 1-point increase in perceived confidence was reported by 46% (*n* = 65) whilst 33% (n = 46) of participants reported a 2- or 3-point increase. No change in perceived confidence was reported by 17% (*n* = 24) whilst a small number of participants (2%, *n* = 3) reported feeling less confident in their skills after completing the training.

Self-reported confidence in managing aggression increased significantly for all participants immediately following the training. Hospital staff who had not been exposed to clinical aggression prior to undertaking the training reported the greatest increase in perceived confidence post-training. In particular, graduate nurses reported the greatest increase in perceived confidence. Staff, other than graduate nurses, who had not been exposed to aggression and therefore not exposed to experiential learning, reported a greater increase in perceived confidence than those staff who had experienced aggression (Table 3). Paired *t* tests were used to compare the difference in mean confidence levels pre- and post-training. *P* values for all groups were < 0.01 indicating strong evidence to reject the null hypothesis, suggesting that training effects were maintained up to 6 months post-training.

**Table 3:** Self-reported confidence levels of staff pre- and post-simulation training in managing clinical aggression

How confident do you feel managing clinical aggression?		*n*	Pre-training		Post-training		Difference (post-pre)		
			Mean (SD)	95% CI	Mean (SD)	95% CI	Mean (SD)	95% CI	*P* value
**All participants**		138	2.49 (0.88)	(2.34, 2.63)	3.66 (0.71)	(3.54, 3.78)	1.17 (0.90)	(1.32, 1.02)	< 0.01
**Experienced aggression**		95	2.62 (0.88)	(2.44, 2.80)	3.67 (0.75)	(3.52, 3.83)	1.05 (0.96)	(1.25, 0.86)	< 0.01
	*GNP*	24	2.25 (0.85)	(1.89, 2.61)	3.5 (0.66)	(3.22, 3.78)	1.25 (1.11)	(1.72, 0.78)	< 0.01
	*Other*	71	2.75 (0.86)	(2.54, 2.95)	3.73 (0.77)	(3.55, 3.92)	0.99 (0.90)	(1.20, 0.77)	< 0.01
**Not experienced Aggression**		43	2.19 (0.82)	(1.93, 2.44)	3.63 (0.62)	(3.44, 3.82)	1.44 (0.67)	(1.65, 1.24)	< 0.01
	*GNP*	30	2 (0.74)	(1.72, 2.28)	3.53 (0.63)	(3.3, 3.77)	1.53 (0.57)	(1.75, 1.32)	< 0.01
	*Other*	13	2.62 (0.87)	(2.09, 3.14)	3.85 (0.55)	(3.51, 4.18)	1.23 (0.83)	(1.73, 0.73)	< 0.01

*GNP* Graduate Nurse Program registered nurse

At completion of the simulation training, participants felt most competent in the use of de-escalation techniques and maintaining patient safety. The largest gains in self-perceived competence as a result of completing the training were in hands on and off restraint and being a group leader (Table 4).

**Table 4:** Self-reported competence in aggression management skills

How competent do you feel displaying these skills when faced with clinical aggression?	Pre-training (***N*** = 140)			Post-training (***N*** = 140)			Follow-up, 3–6 months post-training (***N*** = 44)		
	*n*	Mean (SD)	95% CI	*n*	Mean (SD)	95% CI	*n*	Mean (SD)	95% CI
Being a group leader	138	2.01 (1.11)	(1.82, 2.20)	137	3.10 (1.06)	(2.92, 3.28)	44	3.00 (1.01)	(2.7, 3.3)
De-escalation communication techniques	138	2.80 (0.84)	(2.66, 2.94)	139	3.73 (0.76)	(3.60, 3.86)	44	3.73 (0.79)	(3.50, 3.96)
Maintaining patient safety	139	2.91 (0.86)	(2.77, 3.05)	139	3.88 (0.71)	(3.76, 4.00)	44	4.00 (0.68)	(3.8, 4.2)
Hands off restraint	139	2.33 (1.05)	(2.16, 2.50)	135	3.47 (0.92)	(3.31, 3.63)	44	3.34 (0.83)	(3.09, 3.59)
Hands on restraint	139	2.15 (1.22)	(1.95, 2.35)	136	3.25 (0.96)	(3.09, 3.41)	44	3.07 (0.10)	(3.04, 3.10)
Administering chemical restraint	136	2.26 (1.32)	(2.04, 2.48)	130	2.80 (1.24)	(2.59, 3.01)	44	2.66 (1.23)	(2.30, 3.02)

When asked about confidence to face clinical aggression in the future after the training, 80% of participants indicated that they thought they would be able to manage the situation as a result of completing the simulation training. Whilst no participants reported that they felt unable to manage aggression, 20% remained unsure of their ability to manage clinical aggression in the clinical environment.

### Perceived confidence 3–6 months after training

Participants were asked to rate their perceived confidence levels in managing clinical aggression 3–6 months after the training. The response rate was 31% with 44 respondents completing the online survey. The respondents were representative of the larger group with a similar mix of graduate nurses, ward nurses and senior nurses (Table 2). The follow-up participants had similar levels of clinical aggression experience and the group included a similar mix of mental health versus non mental health staff.

At the 3–6 month follow-up, participants reported reasonable perceived confidence in managing aggression in their clinical environment. The mean perceived confidence score for follow-up participants (3.66, SD 0.71, CI 3.44, 3.88) was similar to perceived confidence immediately following training (3.66, SD 0.71, CI 3.54, 3.78). Those participants who had experienced clinical aggression reported an increased mean perceived confidence score at follow-up (3.71, SD 0.55. CI 3.48, 3.94) compared to immediately post-training. Participants who had not experienced aggression reported similar but lower mean perceived confidence scores at follow-up (3.58, SD 0.90, CI 3.14, 4.01).

Self-reported confidence at follow-up was maintained when compared with perceived confidence levels immediately post-training.

### Reported experience 3–6 months after training

Since attending the simulation training, 66% indicated that they had successfully managed clinical aggression, whilst the remaining 34% had not encountered clinical aggression since undertaking the training. At follow-up, staff who had experienced aggression since completion of the training reported improvements in perceived confidence in managing aggression. Graduate nurses who had not been exposed to aggression also reported further increases in perceived confidence compared to immediately post-training (Table 3).

## Discussion

This is the first study to report development and evaluation of simulation training for paediatric acute care hospital staff in the management of adolescent aggression. Simulation training for this environment was considered an acceptable and worthwhile method of training and staff reported increased perceived confidence in managing clinical aggression following completion of the simulation training with perceived confidence levels maintained for up to 6 months. At follow-up, all participants reported being able to manage clinical aggression. The simulation scenario, with use of a professional actor as the simulated patient, was identified by participants to be an acceptable form of training for the management of clinical aggression. This is consistent with findings of studies of nurses undertaking simulation training for the development of non-technical skills with some studies finding not only was the format acceptable, it was also the preferred method of learning [57–60].

The opportunity to rehearse and practice skills in an environment which closely resembles reality and develop critical thinking and clinical reasoning in a complex care situation without placing patients at risk is highly valued [25, 61] as is the importance of participating in simulated team training [62]. In addition, simulation provides opportunity for exposure to more challenging clinical interactions which clinicians are expected to manage competently despite having infrequent exposure [63].

This study confirmed that staff self-reported confidence levels in managing clinical aggression can be increased through completion of a 2-h simulation training programme and adds to the body of literature that simulation training programmes can improve non-technical skills in health care staff: staff confidence [64], communication skills [33, 65], leadership skills [66–68], team skills [28, 69, 70], knowledge acquisition and retention [71] and skills in aggression management [37, 38, 61, 72]. The use of a simulated patient exercise to practice de-escalation techniques has been described in some acute care hospitals [12, 18, 20, 21, 61] and for undergraduate nursing students [37, 38]; however, widespread use of simulation courses for managing aggression in children and young people in acute health care, and in particular paediatric settings, has not been reported until this study. This proof of concept study provides evidence that simulation training is an acceptable and effective format to rehearse and practice de-escalation skills in the management of clinical aggression.

Newly registered clinicians (Graduate Nurse Program participants in this study) reported the greatest increase in perceived confidence levels in managing clinical aggression following completion of the training. The graduate nurses undertook the simulation training within the first month of their employment with most reporting minimal to no exposure to clinical aggressive incidents prior to the training. It was expected with minimal prior experience in managing aggression that their perceived confidence levels would be low prior to the training and increase following. This finding is consistent with other studies examining the theory–practice gap amongst newly qualified nurses. Simulation exercises in conjunction with other role modelling strategies have been found to reduce transitional stress, increase preparedness for practice and clinician confidence [73–75].

Decline in knowledge and skills following simulation training for health care providers has been well documented [76–78] with skills diminishing faster than knowledge [79]. We found that of the 33% of respondents at 3–6 months follow-up, most reported they retained knowledge and perceived confidence. Two thirds of respondents reported they could manage aggression at follow-up. Confidence in patient management has been shown to be maintained in a number of simulation studies [75, 77, 78, 80] and even increased [81, 82] up to 1 year post-training. This study supports and extends the findings of de la Fuente and Schoenfisch [83] who reported nurses’ confidence in coping with patient aggression to be higher in post behaviour management training and maintained up to 1 month following the training. Nursing staff from an adult medical unit who participated in an interactive programme utilising role plays for managing disruptive patient behaviours reported higher levels of knowledge, attitudes and confidence in managing disruptive at 3 months and 1 year post-training [19]. These studies indicate that self-reported knowledge and skills in aggression management can be maintained up for a year post-training. What is not clear in the literature is whether the use of simulation training is more effective at improving knowledge and skills than other commonly used training formats such as lectures or written or online learning packages. Organisational culture may influence the delivery, uptake and effectiveness of simulation-based group training programmes. Whilst there is evidence that simulation may be the most effective format for teaching and practicing clinical skills [76, 84, 85], there is also evidence that institutional culture, with an emphasis on collaborative, interprofessional learning through simulation coupled with repeated exposure to clinical events, may increase confidence in managing clinical situations long term [82]. This concept is reinforced by this study where participants who had experienced clinical aggression post-training, reported increased perceived confidence at follow-up compared to immediately post-training. Another opportunity for skill maintenance is for simulation interventions to be followed by refresher courses. Refresher courses and adjustments to content delivery have been shown to have the greatest impact on skills retention in a systematic review of RCTs for structured resuscitation training [84].

Participants in our study reported being most competent in maintaining patient safety and de-escalation following training, and this was maintained at follow-up. Participants reported lower competence in being a group leader and implementing restraint which declined at follow-up. Our set of simulation scenarios were primarily focussed on the utilisation and practice of de-escalation skills and maintaining patient safety. Physical restraint is usually utilised when de-escalation methods are unsuccessful and/or patient or staff safety is threatened. The scenario was ceased prior to necessitating 5-point physical or chemical restraint to protect the simulated patient’s safety. This simulation training programme was not designed to focus on using restraint as a first line measure but rather preventative aspects of aggression management, with implementation of a repertoire of de-escalation techniques individually selected and adapted according to the changing clinical situation. If health care organisations consider leadership and competent use of physical and chemical restraint to be key skills for acute care staff, the simulation scenarios can be adjusted, or new scenarios developed, and trialled with these key learning objectives in mind.

### Challenges experienced

Delivering this innovation to nine different training groups enabled us to identify challenges and develop and implement mitigation strategies to improve consistency in experience. One challenge noted was that of participant anxiety. Participants frequently verbalised performance anxiety prior to the simulation sessions even if they had prior simulation experience. To ameliorate anxiety, simulation staff, in a briefing prior to all simulation sessions, provided reinforcement that the experience is a confidential space for learning and reflection. Heightened anxiety was likely due to the large number of junior staff participating or a lack of experience with simulation-based education. In future, we plan to assess the quality and impact on anxiety of our pre-briefing session.

Due to the large number of staff attending each training session, not all participants were able to participate in the simulation scenario and not all participating had key roles during the simulated scenario. Only one participant per scenario could assume the role of group leader so most participants did not practise group leadership skills. However, all participants were able to contribute during the debrief, whether or not they participated in, or observed the scenario, to enable reflection and learning. The debrief at the end of each session addressed scenario objectives and dissected the issues that evolved for this group and the actor. Leadership skills were addressed, and actor feedback and coaching informed the subsequent training sessions to contribute to group learning. Another additional future improvement will be to provide a learning tool that is relevant to the content, for observers to complete whilst watching the simulated scenario [86].

### Limitations

This study was a proof of concept study conducted in one acute care paediatric hospital and as such, the authors did not intend to demonstrate the intervention would have a significant impact on practice. Rather, the aim was to assess if this training innovation is acceptable to participants with the view to design more complex simulations involving young people with autism spectrum disorder and aggressive behaviours and evaluate the effectiveness of this training for acute care paediatric hospital staff in a future trial. Randomised controlled trials which involve multiple sites will provide greater evidence about the effectiveness of this training format. Simulation programmes delivered by trained simulation educators can be replicated in numerous settings to provide larger data sets and stronger evidence in terms of clinical outcomes [28, 87–89].

Only 33% of study participants completed the follow-up survey. It was a purposeful decision to use paper-based pre- and post-surveys for the training groups to ensure optimal response rate. It was not practical to deliver a paper-based follow-up survey to participants located in various departments and wards of the organisation, so an electronic survey was sent. It was anticipated that response rates would be lower with this method as staff may not read emails and may have left the organisation. We cannot be confident that the results of follow-up are true for the whole group. Whilst we were able to perform paired *t* tests on the pre- and post-training group mean perceived confidence scores, this analysis was unsuitable for the post- and follow-up group, as the two samples were not independent and unable to be linked due to anonymity. The differences in mean perceived confidence scores between the post- and follow-up group were, therefore, presented in a descriptive manner.

The design of the simulation scenarios focussed on supportive communication strategies and de-escalation skills rather than the use of major physical and chemical restraint. The use of two-point, upper body restraint techniques was allowed in these simulation scenarios as they posed minimal risk of injury to the actor. Participants rated their perceived confidence in managing full body and chemical restraint lower than other aggression management strategies as expected given the limitation of not including this content in the simulation exercise.

Whilst the majority of participants were nursing staff, with a minority including security, allied health and medical staff, this is a true reflection of the make-up of the Code Grey response team who manages the episodes of clinical aggression in this paediatric hospital. Future studies could compare training programmes which include a more even distribution of professions when appropriate for their hospital setting.

All participants completed the didactic training sessions on aggression management for 6 h prior to commencing the simulation-based group training. We did not assess perceived confidence prior to commencing the training day, which is a limitation of this study. Self-reported confidence levels were, however, reported immediately prior to and following the simulation. In considering the impact of this simulation training, this study utilises the Kirkpatrick Model to evaluate the effect on reaction and learning (levels 1 and 2). Future studies with more comprehensive outcome measures will be able to address the effects on staff behaviour and patient outcomes (levels 3 and 4) [88, 90]. It was the intent of this study to ensure the training format was acceptable for participants and effective in addressing perceived confidence prior to expanding the programme, increasing the scenario complexity and measuring the impact on clinical outcomes. In the future, larger trials are needed that address all four Kirkpatrick levels in order to influence organisational change and demonstrate return on investment of the training.

### Implications for practice and future research

The findings from this study support further development of training programmes to provide education about managing clinical aggression in the paediatric setting, and assessment of effectiveness should be completed. The findings from this proof of concept study may have important implications for a variety of paediatric acute health care settings that are currently experiencing clinical aggression on a daily basis. This study may prompt acute care hospitals to revise their aggression management training to ensure response mechanisms promote physical and psychological safety for staff, and ultimately optimise care for children with neurodiversity and aggressive behaviours.

## Conclusions

Paediatric acute hospital settings are reporting increasing frequency of aggression. Action is needed to support staff to prevent, ameliorate and best manage aggression. This proof of concept study is the first to evaluate a simulation training programme for acute paediatric health professionals on managing clinical aggression in young people. The findings of this study suggest that simulation training for clinical aggression management may be an important addition to staff training programmes in acute care hospitals. Acute care hospitals have a duty to ensure staff are adequately trained and have confidence in hospital systems and processes should aggression be experienced. Further research in this area is warranted, to assess if simulation training will prevent aggressive episodes, reduce patient distress and optimise care through enhancing not only staff confidence, but also competence in managing these patients.

## Data Availability

The datasets used and analysed during the study are available from the corresponding author on reasonable request.

## Abbreviations

MOCA: Management of Clinical Aggression
GNP: Graduate Nurse Program
CNS: Clinical nurse specialist
CNE: Clinical nurse educator
ANUM: Associate nurse unit manager
RN: Registered nurse
MH: Mental health
